# Integration of vector and human behavior in residual malaria in rural communities in the Peruvian Amazon

**DOI:** 10.1371/journal.pntd.0014261

**Published:** 2026-09-25

**Authors:** Carlos Acosta, Diana Cubas-Montecino, Joaquin Gomez, Marlon Saavedra, Mitchel Guzman-Guzman, Sara A. Bickersmith, Jan E. Conn, Dionicia Gamboa, Joseph M. Vinetz

**Affiliations:** 1 Laboratorio ICEMR-Amazonia, Laboratorios de Investigación y Desarrollo, Facultad de Ciencias e Ingeniería, Universidad Peruana Cayetano Heredia, Lima, Peru; 2 Section of Infectious Diseases, Department of Internal Medicine, Yale School of Medicine, New Haven, Connecticut, United States of America; 3 Department of Biomedical Sciences, School of Public Health, State University of New York-Albany, Albany, New York, United States of America; 4 Wadsworth Center, New York State Department of Health, Albany, New York, United States of America; 5 Departamento de Ciencias Celulares y Moleculares, Facultad de Ciencias e Ingeniería, Universidad Peruana Cayetano Heredia, Lima, Peru; 6 Laboratorio de Malaria: Parásitos y Vectores, Laboratorios de Investigación y Desarrollo, Facultad de Ciencias e Ingeniería, Universidad Peruana Cayetano Heredia, Lima, Peru; University of Florida, UNITED STATES OF AMERICA

## Abstract

**Background:**

Malaria persists as a leading cause of morbidity in the Amazon Basin, where the Loreto region of Peru contributes over 90% of the national burden. Despite widespread deployment of long-lasting insecticidal nets (LLINs) and indoor residual spraying (IRS) under Peru’s Malaria Elimination Plan 2022–2030, residual transmission continues in rural riverine communities. This transmission is driven by the interplay between vector ecology and human behavior. This study quantified residual human exposure to *Nyssorhynchus darlingi* by integrating hourly entomological indices with real-time human behavioral data across two seasons in three communities in Mazán, Loreto.

**Methodology/principal findings:**

Entomological (HLC) and epidemiological (HBS) data were collected during the wet and dry seasons of 2022. *Plasmodium* infections were detected in 619 participants by microscopy and qPCR. Molecular surveillance revealed a 13% infection prevalence over seven times higher than routine microscopy (2%) indicating a substantial submicroscopic reservoir. Infection burden was heterogeneous, with Gamitanacocha showing the highest prevalence (33%). LLIN usage was high (>97%), yet nets prevented only 63.3%–80.3% of potential mosquito bites. Residual exposure occurred mainly indoors during early evening (18:00–21:00) while individuals were awake and unprotected, particularly in the dry season when domestic routines coincided with peak vector biting. Outdoor exposure increased during the wet season due to evening recreational activities. Multivariate analysis identified age as the only significant predictor of infection (*p < 0.05*), with individuals over five years showing consistently higher odds of infection than children under five (OR range: 3.62–5.46).

**Conclusions/significance:**

A critical protection gap exists during early evening and dawn, when human activity overlaps with peak vectors biting outside the protection of bed nets. The high submicroscopic burden and persistent indoor exposure while awake demonstrate that current interventions are insufficient for elimination. These findings highlight that Loreto’s elimination strategies must complement intradomiciliary tools with peri-domestic and behavioral interventions—such as full house screening and spatial repellents—specifically targeting the exposure windows identified here.

## Author summary

Malaria persists in the Peruvian Amazon despite high coverage of bed nets and indoor spraying. This “residual transmission” occurs because mosquitoes bite at times and places where current tools offer little protection. We conducted this study in the Mazán district to pinpoint when and where residents are exposed to bites by combining hourly mosquito activity records with real-time surveys of community daily routines.

Our findings reveal a significant “hidden” burden of disease: sensitive molecular testing (qPCR) detected seven times more infections than standard microscopy (13% vs. 2%), identifying a large reservoir of undetected parasites. Although bed net use is near-universal, we found that the greatest risk of being bitten occurs in the early evening (18:00–21:00), when people are active indoors or socializing outside—hours before they go to sleep under their nets.

These results mean that while bed nets remain essential, they cannot eliminate malaria alone because they do not cover the hours of peak human-mosquito interaction. To achieve the goals of Peru’s Malaria Elimination Plan 2022–2030, health programs must look beyond nets, prioritizing home improvements like window screening and developing tools that protect people during their active hours at dawn and dusk.

## Introduction

Malaria is one of the most severe vector-borne diseases worldwide, affecting millions of individuals across 85 endemic countries. In 2022, the global burden reached 249 million malaria cases, an increase of 16 million cases compared to 2019, with Africa accounting for 94% of all infections and 95% of deaths, predominantly among children under five years of age [1]. Although considerable progress has been achieved over the past two decades through large-scale implementation of long-lasting insecticidal nets (LLINs), indoor residual spraying (IRS), and improved diagnostics and treatment, malaria remains a persistent public health challenge.

In the Americas, transmission is concentrated in the Amazon Basin, where ecological, social, and behavioral factors promote the proliferation of *Anopheles* vectors and sustain *Plasmodium* transmission [2]. In Peru, the Loreto region contributes more than 90% of the national malaria burden, making it a critical area for understanding transmission dynamics and for developing integrated control strategies that consider vector ecology, human behavior, and environmental context [3].

Within Loreto, malaria cases are heavily concentrated in the district of Mazán [4]. In 2023, Peru reported 22,349 malaria cases primarily caused by *Plasmodium vivax* (84.37%; 18,856 cases), followed by *Plasmodium falciparum* (15.38%; 3,437 cases) and *Plasmodium malariae* (0.25%; 56 cases). Mazán exhibited one of the highest Annual Parasite Index (API) values in the region, contributing 2.45% of Loreto’s total cases that year. In this district, malaria is predominantly driven by *P. vivax* (74%) and *P. falciparum* (26%).

The main malaria vector in the Peruvian Amazon and in the district of Mazán is *Nyssorhynchus* (also known as *Anopheles) darlingi* [5,6]. Local studies confirm a higher risk of transmission in peridomestic environments than indoors, with marked spatial heterogeneity between and within riverine communities along the Mazán and Napo rivers [7].

Residual malaria transmission refers to the persistence of transmission despite universal coverage with effective LLINs and IRS using insecticides to which local vectors remain susceptible [8]. This phenomenon occurs even where these interventions are widely implemented. Both LLINs and IRS are core strategies in Peru’s Malaria Elimination Plan 2022–2030 and were extensively deployed in Loreto during the Global Fund Project for Malaria Control in Andean Border Areas (PAMAFRO) program (2005–2010) [9,10].

The public health implications of residual malaria transmission are significant. Even under high LLIN/IRS coverage, sustained transmission may prevent elimination unless additional or alternative vector control tools are integrated. This challenge is particularly relevant in rural, forested, riverine, and mobile populations where exposure often occurs outdoors or during hours when insecticide-based interventions provide limited protection.

Malaria risk has traditionally been described using case incidence, parasite prevalence, or measures of transmission intensity and vector abundance [11]. However, a universal definition remains challenging due to the complexity of malaria systems, which involve multiple interacting hosts, vectors, parasites, and environmental variables. Consequently, malaria risk is better conceptualized as a dynamic construct shaped by the presence, density, and infection status of vectors and parasites that vary in space and time. In Mazán’s riverine villages, previous research has shown higher malaria risk in peridomestic areas, with marked spatial heterogeneity between and within communities along the Mazán and Napo rivers [6].

Because rural riverine communities in Mazán face barriers to healthcare access and continue to report a high proportion of malaria cases despite ongoing vector and parasite control efforts, the aim of this study is to investigate residual malaria exposure in three local riverine communities. Specifically, we evaluate exposure during dry and wet seasons to identify gaps in protection under current interventions. By integrating entomological and epidemiological data, this research assesses mosquito exposure adjusted by human behavior and quantifies seasonal variation in *Ny. darlingi* activity. Furthermore, we examine the association between potential risk factors and malaria infection, including human behavior, entomological indices, and indoor-versus-outdoor exposure patterns.

## Results

### Sociodemographic characteristics of the general population

We collected 2,134 records from 619 inhabitants of the three screened communities in six follow-up visits. The male-female ratio was similar across the three communities, with a slightly higher proportion of males in LIB and UM, and the opposite pattern in GAM (Table 1). The age distribution of the study population was also comparable between communities, with 59% (367/619) of participants younger than 30 years, most of them aged 5–15 years. In addition, 40% (249/619) of study participants were engaged in outdoor activities, a proportion that was consistent across communities. Most of the population had primary school education or no formal education (76%; 469/619), although education level differed between communities, with a higher proportion of individuals with secondary education or above in UM (p < 0.001).

**Table 1:** Sociodemographic characteristics of study participants (unique individuals) in the three communities in Mazán.

	Communities			OverallN = 619^1^	p-value^2,3^
	GAMN = 83^1^	LIBN = 256^1^	UMN = 280^1^		
**Sex**					0.11
Female	48 (58%)	114 (45%)	134 (48%)	296 (48%)	
Male	35 (42%)	142 (55%)	146 (52%)	323 (52%)	
**Age (years)**					>0.9
0-5	9 (11%)	21 (8.2%)	26 (9.3%)	56 (9.0%)	
5-15	29 (35%)	79 (31%)	80 (29%)	188 (30%)	
15-30	15 (18%)	47 (18%)	58 (21%)	120 (19%)	
30-50	17 (20%)	55 (21%)	59 (21%)	131 (21%)	
50-65	8 (9.6%)	36 (14%)	37 (13%)	81 (13%)	
65+	5 (6.0%)	18 (7.0%)	20 (7.1%)	43 (6.9%)	
**Primary work**					0.5
Indoor	26 (31%)	67 (26%)	88 (31%)	181 (29%)	
Outdoor	34 (41%)	112 (44%)	103 (37%)	249 (40%)	
Student	23 (28%)	77 (30%)	89 (32%)	189 (31%)	
**Education**					<0.001
None or Primary school	70 (84%)	211 (82%)	188 (67%)	469 (76%)	
Secondary school or higher	13 (16%)	45 (18%)	92 (33%)	150 (24%)	
**Positive Microscopy**					0.024
At least once	3 (3.6%)	7 (2.7%)	1 (0.4%)	11 (2%)	
Not once	80 (96%)	249 (97%)	279 (99.6%)	608 (98%)	
**Positive PCR**					<0.001
At least once	27 (33%)	43 (17%)	9 (3.2%)	79 (13%)	
Not once	56 (67%)	213 (83%)	271 (97%)	540 (87%)	
**Net used previous night**					>0.9
Always used	81 (98%)	247 (96%)	272 (97%)	600 (97%)	
Not used at least once	2 (2.4%)	9 (3.5%)	8 (2.9%)	19 (3.1%)	
**House type**					0.035
Open	13 (16%)	59 (23%)	81 (29%)	153 (25%)	
Closed	70 (84%)	197 (77%)	199 (71%)	466 (75%)	
**House sprayed**					<0.001
No	5 (6.0%)	59 (23%)	127 (45%)	191 (31%)	
Yes	78 (94%)	197 (77%)	153 (55%)	428 (69%)	

^1^N (%) represents the number and percentage of unique individuals (study participants) in each category.

^2^Pearson’s Chi-squared test; Fisher’s exact test.

^3^P-values represent comparisons between communities.

PCR-based detection revealed that 13% (79/619) of participants experienced at least one *Plasmodium spp*. infection during the follow-up period, compared to only 2% (11/619) detected by microscopy, highlighting a substantial underestimation of malaria infections by routine diagnostic methods. Transmission was heterogeneous across communities, with GAM showing the highest proportion of infected individuals (33%, 27/83), followed by LIB (17%, 43/256) and UM (3.2%, 9/280) (p < 0.001). To further characterize infection burden, we quantified the total number of infection episodes across all follow-up visits. A total of 108 Plasmodium infection episodes were detected among the 79 PCR-positive participants. Most episodes were caused by P. vivax (103/108, 95.4%), whereas only three episodes were attributable to P. falciparum (2.8%) and two were mixed-species infections (1.9%). Repeated infections within the same individual were common, particularly in LIB (56/108), followed by GAM (40/108) and UM (12/108). Consistently, GAM exhibited the highest sample-level positivity, with 14% (40/285) of collected samples testing PCR-positive (S3 Table).

Net usage in the sample population was widespread, with only 0.9% (19/2,134) of records reporting not using a net at least once the night before screening (S3 Table). Non-treated and LLINs were present in all communities, but LLINs were preferentially used (83%, 1,781/2,134) over non-treated (16%, 334/2,134) and none (0.9%, 19/2,134). Net ownership and use patterns were similar between communities, and all households reported owning at least one net.

Households were predominantly closed structures (i.e., four walls). Overall, 25% (153/619) of participants lived in open houses (defined as having fewer than four walls), with differences across communities (p = 0.035), being more frequent in UM (29%) and less common in GAM (16%). Indoor residual spraying coverage also varied between communities (p < 0.001), with the highest coverage observed in GAM (94%) and the lowest in UM (55%). Despite these differences, GAM exhibited the highest proportion of PCR-positive individuals, whereas UM had the lowest, suggesting that neither housing structure nor spraying coverage alone explains the observed heterogeneity in malaria across communities (Table 1).

### Entomological characteristics of the study sites

The wet season exhibits a notable increase in mosquito *Ny. darlingi* abundance with significantly greater proportions observed in GAM (93%, N: 4,757) and UM (94%, N: 1,267) compared to LIB (73%, N: 2,441), which has the highest number of mosquitoes in the dry season (27%, N: 918). In addition, a higher proportion of *Ny. darlingi* specimens were collected outdoors in all communities (Table 2). Despite this variation, the proportion of infected mosquitoes or infection rate (IR) remained consistently low. During the wet season, the three communities exhibited an indoors IR of 0.01, while only in the communities of GAM and LIB were infected mosquitoes detected outdoors during the dry season (IR = 0.01). There were no observed seasonal changes in parity indices (S2 Table).

**Table 2:** Summary of *Ny. darlingi* HLC collections by month and community.

Characteristic	GAM	LIB	UM	p-value²^,3^
	N = 5,119¹	N = 3,359¹	N = 1,342¹	
Season				<0.001
Dry	362 (7%)	918 (27%)	75 (5.6%)	
Wet	4,757 (93%)	2,441 (73%)	1,267 (94%)	
Collection site				0.2
Indoors	1,974 (39%)	1,264 (38%)	545 (41%)	
Outdoors	3,145 (61%)	2,095 (62%)	797 (59%)	

¹Mosquito Counts

²Pearson’s Chi-squared test

^3^P-values represent comparisons between communities.

The peak Human Biting Rates (HBRs) were observed during the wet season, predominantly outdoors, with activity typically peaking around 18:00, diminishing overnight, and resurging in the morning. Additionally, multiple peaks were evident in GAM during the wet season, while both GAM and LIB exhibited multiple peaks during the dry season (Fig 1). Compared to UM (Napo River), GAM and LIB (Mazán River) showed a higher indoor HBR index in the early hours of the night during the wet season.

**Fig 1:**
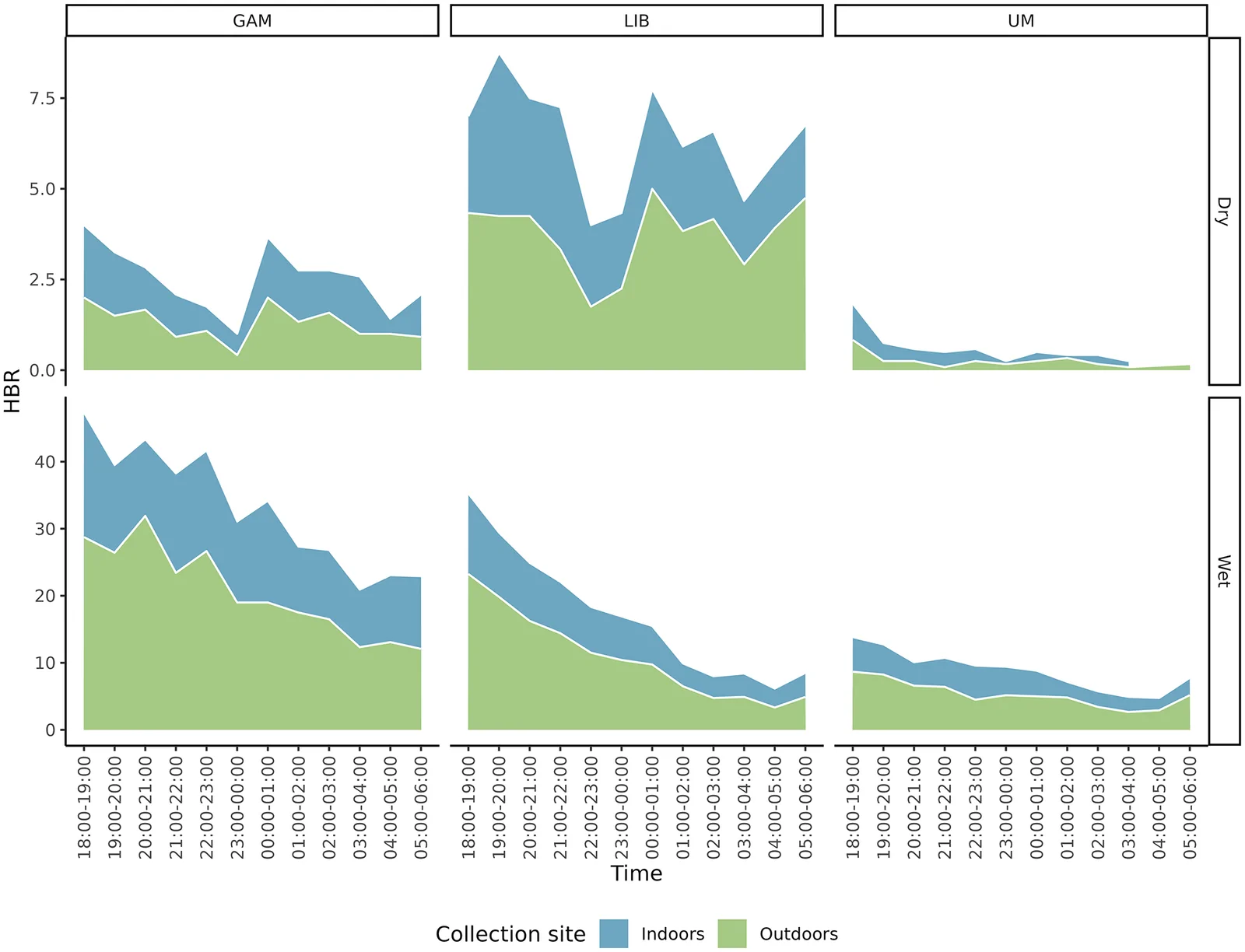
Hourly HBR index of mosquitoes per community and season by collection site (indoors-outdoors).

### *Nyssorhynchus darlingi* exposure in relation to human behavior

Human exposure patterns differ between the dry and wet seasons. During the dry season, a higher proportion of inhabitants remained awake indoors without using long-lasting insecticidal nets (LLINs) during the early evening (18:00–21:00), with the highest values observed between 18:00 and 19:00 (GAM: 66.1%; LIB: 76%; UM: 78.7%). Early-morning outdoor activity was also greater in the dry season, particularly between 05:00 and 06:00 (GAM: 7.8%; LIB: 7.4%; UM: 5%) (Fig 2).

**Fig 2:**
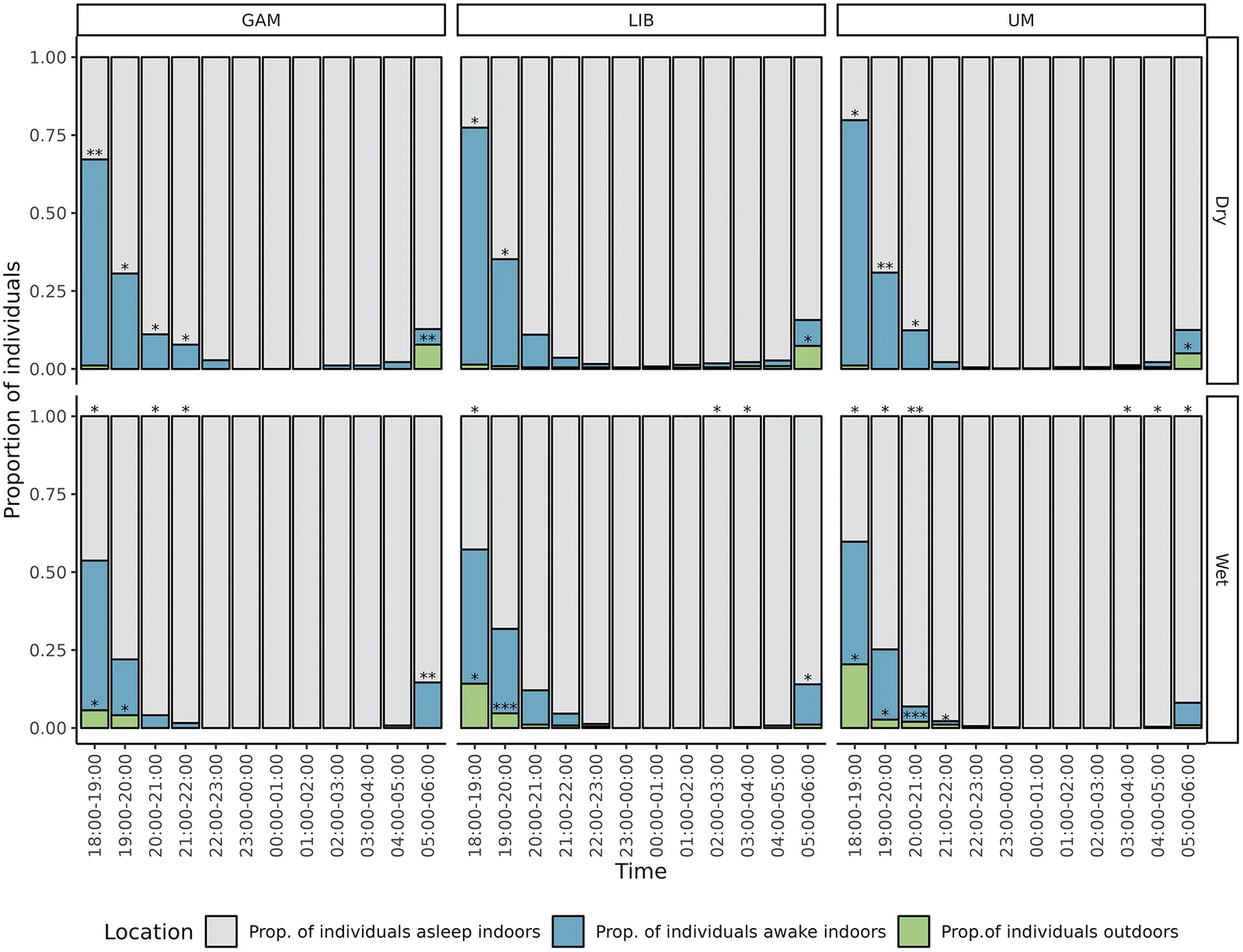
Barplot showing the hourly proportion of individuals indoors, outdoors, and asleep, with significance noted when comparing each of the described proportions between seasons using the proportion test (0 ‘***’ 0.001 ‘**’ 0.01 ‘*’ 0.05).

In contrast, during the wet season, a higher proportion of inhabitants were outdoors during the early evening compared to the dry season. The largest differences were observed between 18:00 and 19:00 (GAM: 11%; LIB: 14%; UM: 11%), with consistently higher outdoor activity across the 18:00–21:00 period.

During the dry season, a higher proportion of individuals were active outdoors in the early morning (05:00–06:00 h), mainly due to work-related activities (S3 Fig). In the early evening (18:00–19:00 h), bathing—often conducted outdoors or in semi-open structures—was frequently reported and coincided with a peak in vector biting activity (S4 Fig). In contrast, during the wet season, early-evening outdoor recreational activities were more frequently reported (S5 Fig), overlapping with periods of high vector density.

By integrating hourly entomological data with human behavior patterns, we estimated potential *Ny. darlingi* biting exposure and quantified LLIN protection in the Mazán district. LLINs prevented 63.31% to 80.25% of potential exposure depending on community and season (Fig 3). Residual exposure occurred primarily indoors while individuals were awake during the dry season (GAM: 62.12%, LIB: 56.52%, UM: 79.39%), with the highest values observed during the early evening hours (18:00–21:00). In the wet season, outdoor residual exposure increased (GAM: 9.1%, LIB: 22.83%, UM: 23.84%) and was also concentrated in the early evening, while indoor exposure while awake remained predominant in GAM and LIB.

**Fig 3:**
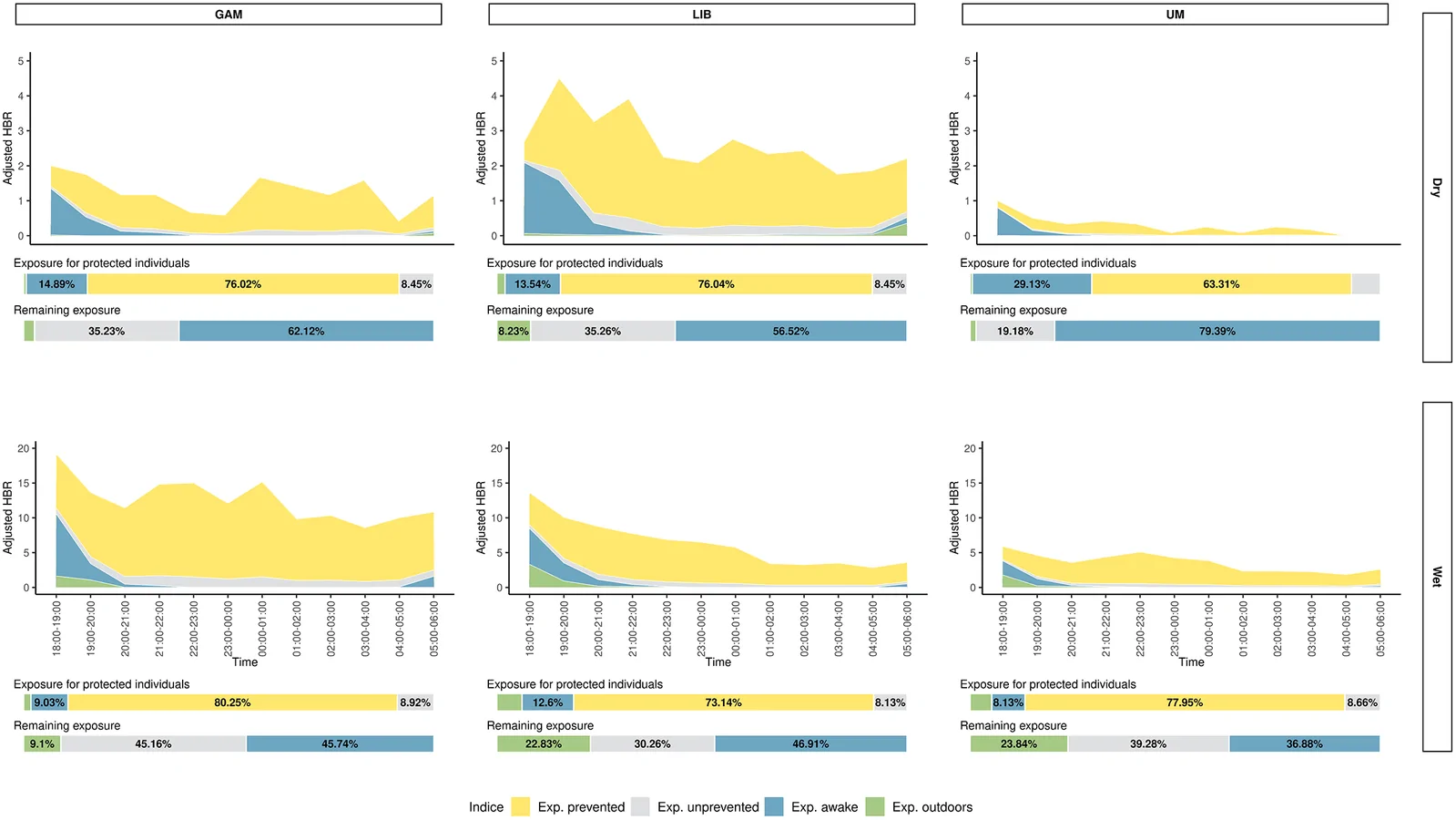
Adjusted hourly HBRs per season and community. The exposure and the remaining exposure for protected individuals are shown as barplots.

### Factors associated with *Plasmodium* spp. infection

Mixed-effects univariate logistic regression analyses identified age as the only factor consistently associated with *Plasmodium spp*. infection (Table 3). Compared with children aged 0–5 years, all older age groups had significantly higher odds of infection (OR range: 3.6–5.6; all p < 0.05). No significant associations were observed for sex, primary work location, indoor HBR, indoor or outdoor EIR, indoor residual spraying (IRS), net type used the previous night, or hours spent under a long-lasting insecticidal net (LLIN) (all p > 0.05). Outdoor HBR showed a non-linear pattern, with the highest exposure category (>50–140 bites/person/night) associated with lower odds of infection compared with the reference category (OR = 0.62, 95% CI: 0.38–1.00).

**Table 3:** Univariate and Multivariate mixed models testing the association of different factors to qPCR positive *Plasmodium* spp. infection. Variables for the multivariate model were selected by AIC.

Characteristic	Univariate model			Multivariate model		
	OR^1^	95% CI^1^	p-value	OR^1^	95% CI^1^	p-value
Age (years)						
0-5	—	—		—	—	
5-15	3.61	1.10, 11.9	0.035	3.62	1.09, 11.9	0.035
15-30	4.02	1.17, 13.9	0.028	4.07	1.18, 14.1	0.027
30-50	5.55	1.67, 18.4	0.005	5.46	1.64, 18.2	0.006
50-65	3.84	1.07, 13.7	0.038	3.75	1.05, 13.5	0.042
65+	4.89	1.29, 18.5	0.020	4.97	1.31, 18.9	0.019
Sex						
Female	—	—				
Male	1.16	0.78, 1.73	0.5			
Primary work						
In	—	—				
Out	1.43	0.89, 2.29	0.14			
Student	0.90	0.52, 1.55	0.7			
HBR indoors						
<5	—	—				
>5-20	1.17	0.37, 3.69	0.8			
>20-40	0.74	0.23, 2.39	0.6			
>40	0.66	0.20, 2.25	0.5			
HBR outdoors						
<20	—	—		—	—	
>20-50	1.39	0.80, 2.43	0.2	1.35	0.77, 2.37	0.3
>50-140	0.62	0.38, 1.00	0.049	0.62	0.38, 1.01	0.053
EIR indoors						
No EIR	—	—				
>0-0.5	0.47	0.19, 1.15	0.1			
>0.5-1.0	0.94	0.43, 2.08	0.9			
EIR outdoors						
No EIR	—	—				
>0-1.0	0.91	0.58, 1.41	0.7			
>1.0-3.0	0.68	0.31, 1.51	0.3			
House sprayed						
No	—	—				
Yes	0.72	0.43, 1.19	0.2			
Net used previous night						
Not used	—	—				
Untreated net	0.95	0.55, 1.62	0.8			
LLIN	1.01	0.13, 7.84	>0.9			
Time under LLIN last night	0.98	0.87, 1.10	0.7			
community.sd__(Intercept)				1.385		

^1^OR = Odds Ratio, CI = Confidence Interval.

In the final multivariable mixed-effects logistic regression model (Table 3), age was the only statistically significant variable. Compared with children aged 0–5 years, all age groups older than 5 years had significantly higher odds of malaria infection, with adjusted ORs ranging from 3.62 to 5.46. Outdoor HBR was not significantly associated with infection risk; however, the highest exposure category (>50–140 bites/person/night) showed a borderline association with lower odds of infection (OR = 0.62, 95% CI: 0.38–1.01; p = 0.053).

## Discussion

The present study addresses the need for precise data by quantitatively integrating the hourly activity of *Ny. darlingi* with detailed patterns of human exposure in Amazonian riverine communities. Our findings reveal that seasonal changes in human behavior create temporal mismatches between vector activity and LLIN use, generating periods of increased exposure. In particular, exposure occurs during early evening and dawn hours, when individuals are active but not yet protected by LLINs, consistent with the concept of a “protection gap” described in other endemic settings [12–14]. These patterns indicate that human behavioral rhythms play a central role in shaping seasonal transmission dynamics beyond what is captured by vector density alone.

Saavedra et al. [6] provide one of the earliest integrative entomological–epidemiological assessments for Mazán, offering an important baseline for communities comparable to LIB and UM. In contrast to the biting pattern, they reported for 2016–2017, where *Ny. darlingi* activity gradually increased after 18:00 and peaked around 21:00, our 2022 data from LIB and UM indicate that high biting activity was already present at 18:00, followed by a gradual decline later in the night. Although the sampling methodology was comparable between studies, it is important to note that the earlier study included four communities, whereas the 2022 survey included three, with only two communities overlapping between both study periods. Therefore, part of the observed difference in biting patterns may reflect spatial variation in vector behavior between communities. In addition, the earlier concentration of biting activity may also reflect interannual variation in ecological conditions, changes in host availability during early evening hours, or behavioral responses to sustained vector control pressure such as LLIN use and indoor residual spraying. These findings highlight the plasticity of *Ny. darlingi* biting behavior and the importance of considering spatial and temporal heterogeneity when evaluating residual malaria transmission.

Our findings regarding residual exposure align with observations from Namibia [15], where human behavior substantially shaped malaria exposure patterns despite high LLIN coverage. We observed high levels of indoor residual exposure while individuals are awake, particularly in UM during the dry season. This highlights a critical protection gap that LLINs alone cannot mitigate, as residents are present indoors but not yet under a net. Although indoor residual spraying (IRS) coverage varied substantially between communities (Table 1), ranging from 55% in UM to 94% in GAM, its epidemiological impact was difficult to detect in our analyses. In the univariate model, IRS was associated with a lower odds of *Plasmodium spp*. infection (OR = 0.72, 95% CI: 0.43–1.19), although this association did not reach statistical significance and the variable was not retained in the final multivariable model following AIC-based selection. This finding should not be interpreted as evidence of an absent IRS effect, but rather as a reflection of the multiple factors that can obscure its detection in residual transmission settings. First, the protective effect of IRS depends strongly on the timing of spraying campaigns relative to peak transmission periods, the completeness of household coverage, and the persistence of residual insecticidal activity on sprayed surfaces throughout the season. Second, *Ny. darlingi* exhibits considerable behavioral plasticity, including exophilic resting behavior after blood feeding [6], which may reduce mosquito contact with insecticide-treated indoor surfaces and limit the efficacy of IRS as currently implemented. Finally, as discussed above, the predominance of *P. vivax* infections—many of which may represent relapses from hypnozoites activated months after the original infectious bite—likely weakens the temporal association between contemporary vector control interventions, including IRS, and observed infection outcomes. Taken together, these factors highlight that the limited statistical detectability of an IRS effect in this study reflects methodological and biological constraints rather than a true absence of protection and underscore the need for spatially and temporally resolved evaluations of IRS effectiveness in future residual transmission studies. In this context, the implementation of screened houses represents a promising complementary intervention. Several studies have documented significant reductions in indoor mosquito entry using screening [16–18], along with evidence of high community acceptability [19]. The residual exposure in Mazán, concentrated in the early evening (18:00–21:00) across all three communities, further emphasizes the potential of targeted behavioral interventions or supplementary vector control strategies during this period. Similarly, qualitative research in Zanzibar found that evening social, recreational, and livelihood activities were major barriers to timely insecticide-treated net use, reinforcing the need for strategies that consider human behavior alongside conventional vector control tools [20].

Despite similar net-use patterns across Mazán communities, mosquito abundance differed significantly between seasons, with higher densities in the wet season, yet malaria incidence peaked in the dry season (S2 Fig). This pattern, previously observed in Brazil [21], may result from behavioral exposure mechanisms, including increased dawn activity and evening hygiene routines. While bed net usage did not differ significantly across Mazán communities (Table 1), the seasonal variation in mosquito abundance highlights the complex interplay between vector density, human behavior, and infection risk.

The mixed-effects logistic regression indicates that age was the strongest predictor of malaria infection risk in the studied communities, while entomological indicators and preventive behaviors were not significantly associated with infection risk in the final model. Although the AIC-based model selection included age and HBR outdoors, as covariables, only age was statistically significant, suggesting that demographic factors explained a larger proportion of the observed variability in infection risk than the available entomological and behavioural indicators. The association between increased age and malaria risk aligns with previous findings from Amazonian Peru, where older age groups showed higher malaria incidence [5]. This may reflect cumulative exposure over time, shifts in occupational roles—particularly toward forest-based activities—or age-dependent differences in acquired immunity [22,23]. Although the mixed-effects regression did not show a statistically significant association between entomological indices and malaria at the global level—likely reflecting the combined influence of temporal lags, spatial heterogeneity in transmission, and the limited resolution of community-level entomological estimates—visual trends reveal clear season-dependent biological patterns (S1–S2 Figs).

During the wet season, increases in the Human Biting Rate (HBR)—both indoors and outdoors—were accompanied by higher malaria prevalence in Mazán basin communities, consistent with literature linking rainfall with increased vector density. These communities also exhibited the highest HBR, EIR, incidence, and prevalence compared with the Napo basin, consistent with previous findings [6]. A notable finding was the peak in malaria prevalence during the dry season across all three communities, despite low HBR and EIR. This apparent mismatch is consistent with the temporal lag between infection and clinical detection. According to Reiner Jr. et al. (2015) [24], malaria seasonality is a complex phenomenon where climatic factors do not act immediately, but rather through temporal lags that vary significantly depending on the local environment. In the specific context of Peru, it has been observed that increases in malaria cases typically occur between 3 and 16 weeks after entomological peaks or weather events, with a peak incidence ratio at approximately five weeks [25]. Thus, dry season cases likely reflect infections acquired during the preceding wet season vector peak. These heterogeneous patterns underscore the complexity of vector–parasite dynamics and reinforce the need for longer follow-up periods.

An additional consideration is the predominance of *P. vivax* transmission in the study area. Unlike *P. falciparum*, *P. vivax* infections may arise from relapses occurring months after the initial infectious mosquito bite due to activation of dormant liver hypnozoites. Consequently, contemporary entomological conditions and current human behavioral patterns may not accurately reflect the exposure event responsible for a given infection episode. This temporal disconnect may have further reduced our ability to detect associations between vector exposure metrics and malaria risk and may partially explain why age was the only variable consistently associated with infection in the multivariable analyses. Furthermore, because malaria transmission during the study period was overwhelmingly dominated by *P. vivax* (95.4% of infection episodes), whereas only three *P. falciparum* infections and two mixed infections were detected, species-specific analyses were not statistically feasible and would likely have produced unstable estimates with wide confidence intervals.

We also highlight the importance of optimizing HLC sampling strategies. Guzmán et al. (2025) [5] provides evidence that malaria transmission is spatially clustered and that vectors tend to concentrate in degraded forest landscapes. Thus, incorporating prior knowledge of household-level case distribution could help direct sampling toward transmission “hotspots” or increase household coverage, ultimately improving alignment between entomological and epidemiological indicators [26].

Further limitation of this study relates to the low number of infected mosquitoes detected, which may have reduced the statistical power to identify associations between entomological indicators and malaria risk. Malaria transmission in the Peruvian Amazon is highly heterogeneous and spatially clustered, with vectors often concentrating in specific ecological settings, including degraded forest landscapes and peri-domestic environments. In this context, entomological estimates derived from HLC conducted in only four households per community per month, which may not have fully captured the substantial household-level variability in vector exposure characteristic of Amazonian transmission settings. Consequently, community-level HBR and EIR may have limited sensitivity to detect fine-scale associations with individual infection risk.

In addition, malaria transmission is influenced by complex temporal and ecological lag effects, whereby environmental drivers such as river level fluctuations contribute to vector population dynamics and transmission with delayed effects due to mosquito development and parasite incubation periods [24]. Therefore, the relatively short duration of follow-up in this study may have limited our ability to fully capture these lagged transmission dynamics. Future studies incorporating higher spatial sampling resolution and prior knowledge of transmission hotspots could improve the detection of vector exposure heterogeneity and strengthen the alignment between entomological and epidemiological indicators.

This study suggests that residual malaria transmission in the Peruvian Amazon is sustained by a critical spatiotemporal convergence between the plastic biting behavior of *Ny. darlingi* and seasonal human routines that circumvent traditional indoor interventions. The persistence of transmission may be sustained by exposure during early evening and dawn activities—such as bathing and recreation—highlighting a significant protection gap occurring outside periods of optimal LLIN protection. Since age emerged as the strongest predictor of infection in our analyses, our findings underscore that current intradomiciliary strategies may not fully address residual exposure occurring outside sleeping hours. Consequently, effective malaria elimination in these riverine communities urgently requires the integration of complementary tools, such as full mosquito-proof housing (including screened eaves and windows) and spatial repellents, specifically designed to target the indoor and peri-domestic exposure windows identified in this analysis.

## Methods

### Ethics statement

The Amazonian International Center of Excellence for Malaria Research (ICEMR) in Peru collected the data used for this work. Both Epidemiology and Entomology subprojects were reviewed and approved by the Institutional Ethics Committee in Research (CIEI) of the Universidad Peruana Cayetano Heredia (UPCH), under SIDISI codes 101518 and 101516, respectively. Every adult participant (18 years and older) signed a written informed consent form prior to enrollment and blood sample collection. Participants less than 18 years old provided informed verbal assent, along with a parent or guardian signature which documented signed, written, formal informed consent form before enrollment.

### Study area and population

The study was conducted in the Amazonian district of Mazán, Loreto (Fig 4). Loreto is characterized by tropical weather, with temperatures ranging from 18°C to 36°C and an annual precipitation of 2,234 mm. Rainfall is highest from December to May, with an average annual humidity of 84%. The province of Maynas is divided into eleven districts, including Mazán. To define the study periods, time series analysis was performed using river level data from the Mazán (Bellavista) hydrological station, obtained from the Autoridad Nacional del Agua (ANA), Peru, through the *Sistema Nacional de Información de Recursos Hídricos* (SNIRH) [27]. The seasonal component revealed two contrasting hydrological periods: the dry season (September–February) and the wet season (March–July), the latter characterized by a rising phase beginning in March and peaking between May and July. These patterns were used to establish the two sampling periods (S6 Fig).

**Fig 4:**
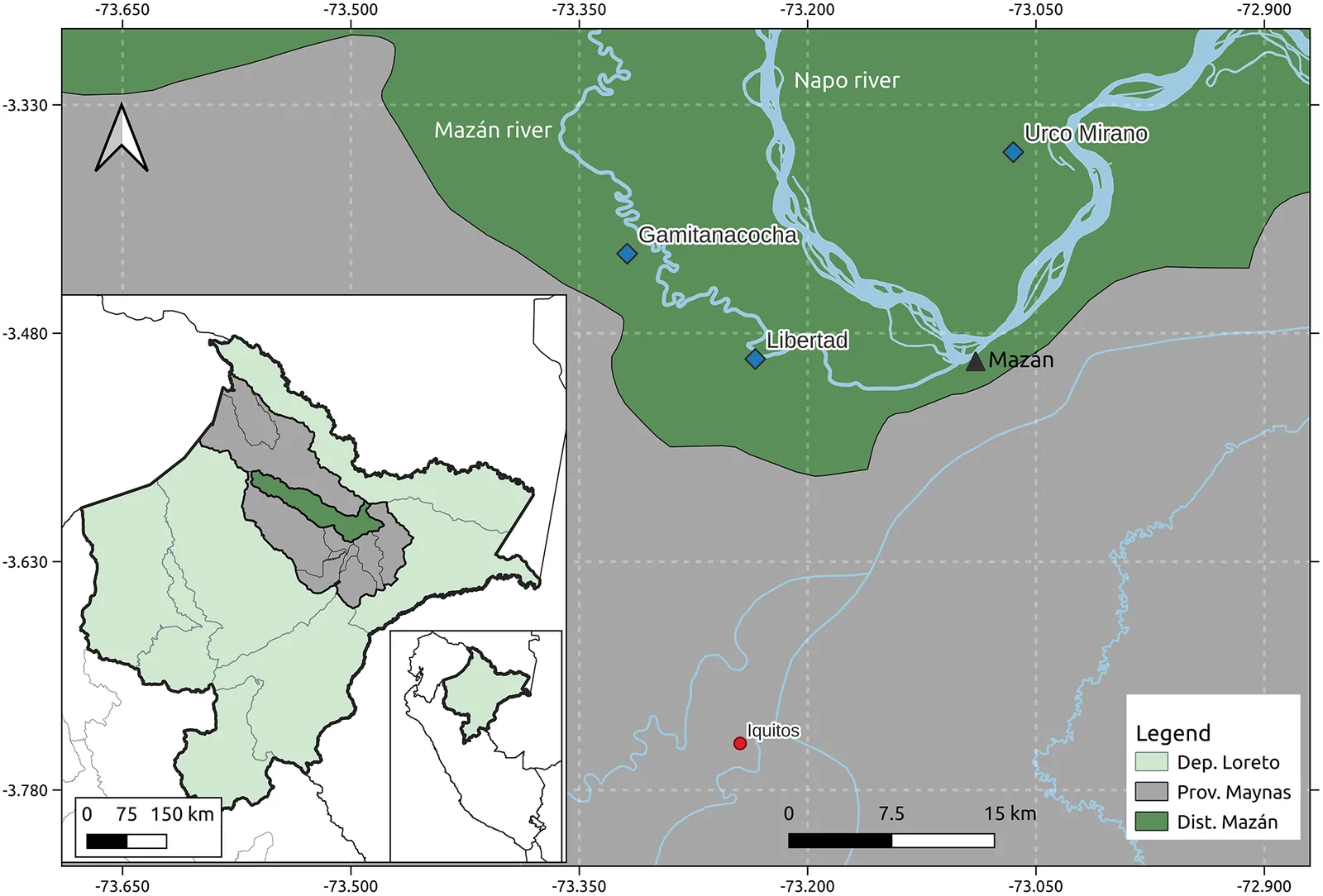
Map showing Gamitanacocha, Libertad, and Urco Mirano communities along the Mazán and Napo rivers, Mazán District, Maynas Province, Loreto Department, Peru. Insets show the administrative context within Peru. The map was generated using QGIS 3.22. (QGIS Development Team, 2023. QGIS Geographic Information System. Open-Source Geospatial Foundation Project. http://www.qgis.org/). Shapefiles are freely available on the Instituto Nacional de Estadística e Informática (INEI) website (https://ide.inei.gob.pe/). No copyrighted basemaps or proprietary cartographic layers were used.

The Mazán district is located approximately 50 km north of Iquitos City (capital of Loreto department). Nearly 30% (~3,700 inhabitants) of the district’s population lives in Mazán town, the district’s capital, while the remaining 70% (~12,300 inhabitants) lives in rural villages dispersed along the Mazán and Napo rivers (Fig 4). The study area was characterized using INEI demographic projections [28] and malaria records from GERESA [29] and open data sources. These datasets were used to calculate the Annual Parasite Incidence (API) for 2018–2025 (S1 Table), identifying a fluctuating pattern with a cyclical resurgence every three years. Additionally, hydrological seasonality was determined via ANA/SNIRH river level data, identifying key phases such as the rising period (March) and the peak (June–July) (S6 Fig).

### Study design and data collection

Entomological and human blood samples were collected by subprojects within the *Amazonian International Centers of Excellence for Malaria Research* program in Peru. Data were collected in three rural Amazonian communities, Libertad (LIB), Urco Mirano (UM), and Gamitanacocha (GAM) (Fig 4), during three months in the wet season (March to May) and three months in the dry season (October to December) (Fig 5). Study site selection was guided by previous extensive research within the area, malaria transmission history, and convenience for sample storage and transportation due to proximity to the city of Iquitos.

**Fig 5:**
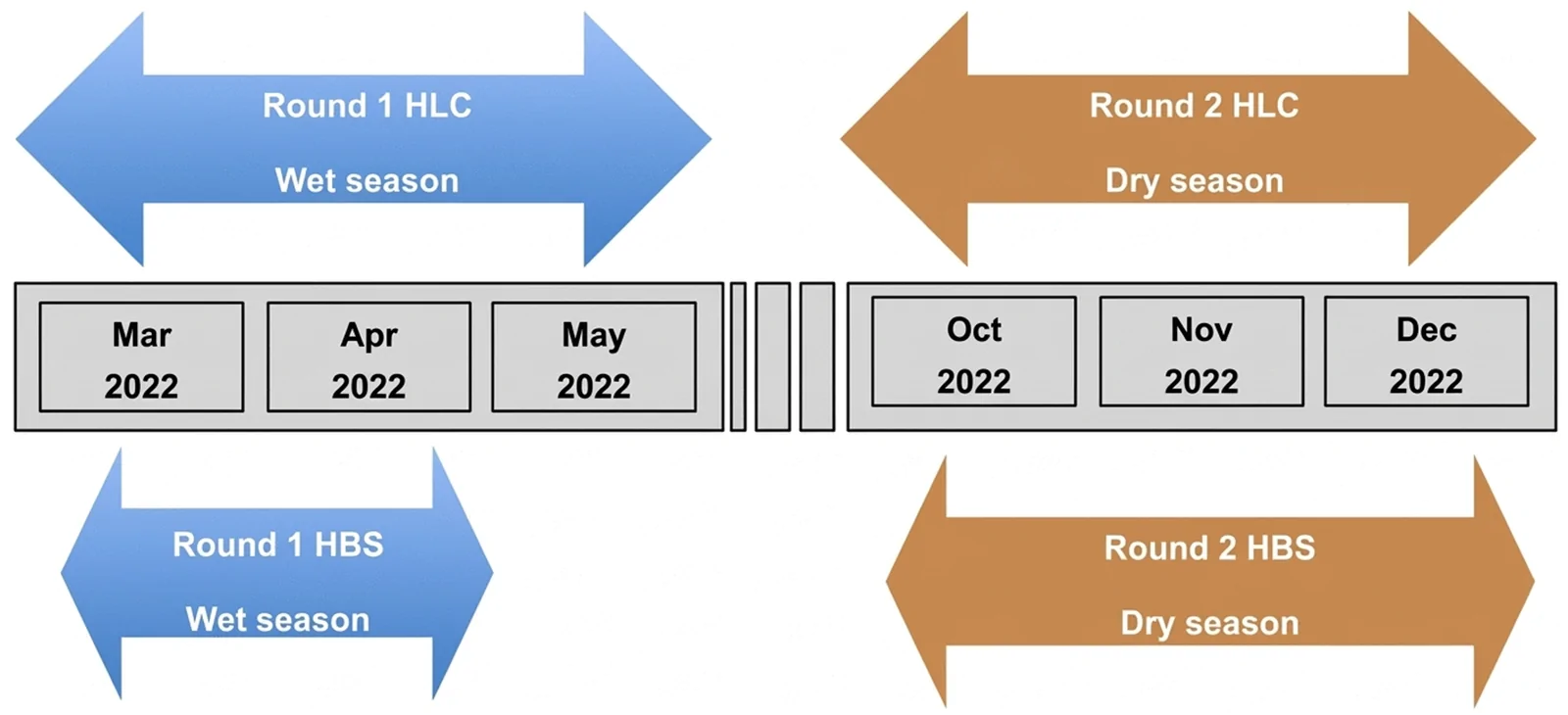
Timeline of human behaviour surveys (HBS) and entomological surveys by Human Landing Catch (HLC).

Prior to data collection, a census was conducted to estimate the total human population and map households and locations for entomological collections. A brief questionnaire deployed in the Open Data Kit (ODK) electronic data capture system and handheld Global Positioning System (GPS) devices (Garmin’s GPSMAP 60CSx, Garmin International Inc., USA) were used during censuses.

The entomological fieldwork was carried out one week before cross-sectional whole-population screenings. Four households were selected randomly each month, and human landing catches (HLC) were conducted for two nights. Two households were sampled per night, with one collector positioned indoors and one outdoors per household, totaling two indoor and two outdoor collectors per sampling night. HLC was performed for twelve hours (18:00 – 06:00 hrs) simultaneously inside and outside, within a range of 3–5 meters of the households. Collected mosquitoes were stored in individual centrifuge tubes with silica gel at room temperature until laboratory analysis.

Epidemiological data collection was carried out within one week after the entomological collection to ensure the closest possible overlap of human and vector behavior data. Questionnaires were designed using the REDCap online tool and employed in the field using the REDCap mobile application (http://www.projectredcap.org/). Individual and household data were collected during each screening, including self-reported human behavior from the previous night (18:00–06:00), LLIN usage patterns, and house characteristics. Finally, blood samples were taken by finger puncture and stored in EDTA microtainers until further processing.

### Laboratory procedures

#### Microscopy.

Blood sample *Plasmodium* spp. infection was assessed using microscopy on the day of collection. Thick and thin smears were prepared with 10% Giemsa solution for 10 minutes. Parasitemia was assessed by counting the number of asexual parasites per 200 leucocytes. Samples were classified as negative if no parasites were found within 100 microscopy fields [30].

#### Real-time Quantitative PCR.

DNA was obtained from whole blood samples using kit commercial according to the manufacturer guidelines, with slight modifications (addition of TEN buffers [20 mM Tris-HCl, pH 8.0; 2 mM EDTA, pH 8.0; 0.2 M NaCl] supplemented with SDS 10% w/v). Subsequently, a quantitative polymerase chain reaction (qPCR) to detect *Plasmodium* spp. was conducted according to Mangold et al. [31]. Positive samples were subsequently analyzed using species-specific real-time PCR assays to identify the infecting Plasmodium species, including *P. vivax* and *P. falciparum*, following the protocol described by Rougemont et al [32]. For the statistical analyses, the outcome variable was defined as PCR-confirmed *Plasmodium* infection irrespective of species.

### Entomological laboratory procedures

#### Detection of *Plasmodium* spp. in mosquitoes.

The head/thorax of specimens collected in the Mazán district were analyzed in pools of two to five individuals (same species and same collection date/time). The ELISA protocol was performed following Wirtz et al. [33] and can distinguish between circumsporozoite protein (CSP) from *P. falciparum* and *P. vivax* (Pv210 and Pv247). The optical density of processed plates was measured at 410 nm in a Bio-Rad ELISA plate reader 30 and 60 minutes after substrate addition. For each ELISA plate, seven negative controls consisting of laboratory-reared mosquitoes that had never been exposed to *Plasmodium*-infected humans were included. The positivity cut-off was defined as twice (2×) the mean OD of these negative controls for each plate, thereby accounting for plate-to-plate variation. Mosquito pools with OD values exceeding the plate-specific cut-off were classified as positive for CSP.

#### DNA extraction and PCR analysis.

Genomic DNA extraction from the abdomen of each mosquito specimen was carried out using the DNeasy tissue kit (Qiagen, CA, USA). Specimens difficult to identify morphologically, such as cryptic *Nyssorhynchus* species or damaged specimens, were identified using the internal transcribed spacer 2 (ITS2) sequence [34], ITS2-RFLP [35], and cytochrome c oxidase subunit I (*COI*) barcode region [36]. *Plasmodium* detection in mosquitoes was performed following the PCR-RFLP protocol described by Hasan et al. [37], specifically targeting the *Plasmodium* cytochrome b (*Cyt-b)* gene. The procedure was performed on the positive ELISA pools to identify the positive sample and as a verification.

#### Estimation of mosquito parity and daily survival rate.

To estimate the age composition of females in the population, a proportion of each month’s collection (approximately 10%) of female mosquitoes was dissected to determine the parity rate per collection month. Parity was also used as an indicator of survival under natural conditions. Mosquito longevity (life expectancy) and daily survival were estimated using the methods described in [38–42].

### Data analysis

#### Entomological indicators.

The human-biting rate (HBR) was calculated as the average number of *Ny. darlingi* bites per collector per hour and plotted by community and season. Additionally, the infection rate (IR) and entomological inoculation rate (EIR) were calculated by month (S2 Table).

#### Indicators of human‑vector interaction patterns.

To characterize temporal patterns of human exposure, we calculated the hour-by-hour proportions of individuals indoors, outdoors, and asleep for each community and season. Seasonal differences in these behavioral patterns were evaluated using proportion tests to identify shifts in human–vector interaction relevant to malaria transmission. Building on these behavioral data, entomological indicators were calculated following the method proposed by Monroe et al. (2020) [12], which estimates the incidence of mosquito bites by combining individual exposure patterns with the number of mosquitoes collected indoors and outdoors. LLIN use was incorporated using a conservative protection factor of 0.9, reflecting the standard assumption in vector exposure and transmission models that sleeping under an LLIN prevents approximately 90% of mosquito bites, as established by [43,44] and previously applied in this region by Rosas-Aguirre et al. [45].

Exposure was summarized using five indices:

- $\pi_{I,\;u}$: Proportion of vector bites occurring indoors for an unprotected individual.
- $\pi_{O,\;u}$: Proportion of vector bites occurring outdoors for an unprotected individual.
- $\pi_{S,\;u}:\;$Proportion of vector bites occurred while an unprotected individual was asleep.
- ${P^{*}}_{s,u^{\prime }}$: Proportion of all vector bites directly prevented by use of an LLIN.
- $\pi_{I}$: Proportion of remaining exposure occurring indoors for a protected user of a LLIN.

These indices were estimated and plotted by season, with a special focus on residual exposure.

#### Statistical analysis.

Generalized linear mixed-effects models (GLMMs) with a binomial error distribution and logit link function were used to evaluate factors associated with Plasmodium spp. infection. The unit of analysis was the screening observation (participant-visit record), corresponding to an individual screened during a specific follow-up visit. Participants could contribute multiple observations across the repeated cross-sectional surveys conducted during the study period. Entomological indicators were assigned according to the community and sampling period corresponding to each screening observation. Community was included as a random intercept to account for clustering of observations within communities. Univariate GLMMs were fitted separately for each explanatory variable. A full multivariable GLMM including all candidate predictors was fitted, followed by backward variable selection based on the Akaike Information Criterion (AIC) to identify the most parsimonious model. Variables were sequentially removed until no further reduction in AIC was achieved. The final multivariable GLMM was subsequently refitted including only the predictors retained after the AIC selection procedure. Effect estimates are presented as odds ratios (ORs) with corresponding 95% confidence intervals (95% CIs).

Because transmission during the study period was overwhelmingly dominated by *P. vivax* and only a small number of *P. falciparum* infections were detected, all regression analyses were conducted using PCR-confirmed *Plasmodium spp.* infection as a binary outcome variable to ensure stable parameter estimation and adequate statistical power.

All statistical analyses were performed using R version 4.3.3 (R Core Team, 2023).

## Supporting information

S1 Table Demographic and malaria incidence in Mazán (2018–2025). (XLSX)

S2 Table Estimation of infectivity rate (IR), human-biting rate (HBR), entomological inoculation rate (EIR), parity status, and life expectancy of *Nyssorhynchus darlingi* across study communities and seasons. (XLSX)

S3 Table Sociodemographic, exposure, and entomological correlates of *Plasmodium* spp. infection status determined by PCR. (XLSX)

S1 Fig Monthly prevalence of microscopic and submicroscopic *Plasmodium* spp. infections and corresponding indoor and outdoor Entomological Inoculation Rates (EIR) across GAM, LIB, and UM communities. (TIFF)

S2 Fig Monthly prevalence of microscopic and submicroscopic *Plasmodium* spp. infections and corresponding indoor and outdoor Human-Biting Rate (HBR) across GAM, LIB, and UM communities. (TIFF)

S3 Fig Outdoor activity between 05:00 and 06:00 across wet and dry seasons. Some values may appear inflated because individuals can perform more than one activity simultaneously; however, individual percentages were used.
(TIFF)

S4 Fig Indoor activity between 18:00 and 19:00 across wet and dry seasons. Some values may appear inflated because individuals can perform more than one activity simultaneously; however, individual percentages were used.
(TIFF)

S5 Fig Outdoor activity between 18:00 and 19:00 across wet and dry seasons. Some values may appear inflated because individuals can perform more than one activity simultaneously; however, individual percentages were used.
(TIFF)

S6 Fig Time series decomposition (trend, seasonal, and residual components) of monthly water level in the Napo River at Bellavista hydrological station, Peruvian Amazon (2017–2020). Data were obtained from the Autoridad Nacional del Agua through the Sistema Nacional de Información de Recursos Hídricos (SNIRH) platform.
(TIFF)
